# A contribution to the taxonomy, cytogenetics and reproductive biology of the genus *Aclerda* Signoret (Homoptera, Coccinea, Aclerdidae)

**DOI:** 10.3897/CompCytogen.v6i4.4320

**Published:** 2012-11-30

**Authors:** I.A. Gavrilov-Zimin

**Affiliations:** 1 Zoological Institute, Russian Academy of Sciences, Universitetskaya nab. 1, St. Petersburg, 199034, Russia

**Keywords:** Scale insects, Aclerdidae, *Aclerda takahashii*, *Aclerda pseudozoysiae*, taxonomy, morphology, new species

## Abstract

A new species of scale insects, *Aclerda pseudozoysiae* **sp. n.,** is described and illustrated. The karyotypes and some aspects of reproductive biology and cytogenetics of the new species species and *Aclerda takahashii*Kuwana, 1932 were studied, representing the first data for the genus *Aclerda* Signoret, 1874 and the family Aclerdidae as a whole. *Aclerda pseudozoysiae* **sp. n.** has 2n=16, bisexual reproduction, and heterochromatinization of one haploid set of chromosomes in male stages of the life cycle, matching either a Lecanoid or a Comstockioid genetic system. *Aclerda takahashii* demonstrates 2n=18 and unusual type of parthenogenesis with diploid and haploid embryos (inside each gravid female) without heterochromatinization. Both species are ovoviviparous; all stages of embryonic development occur inside the mother’s body.

## Introduction

**Figure 1.**
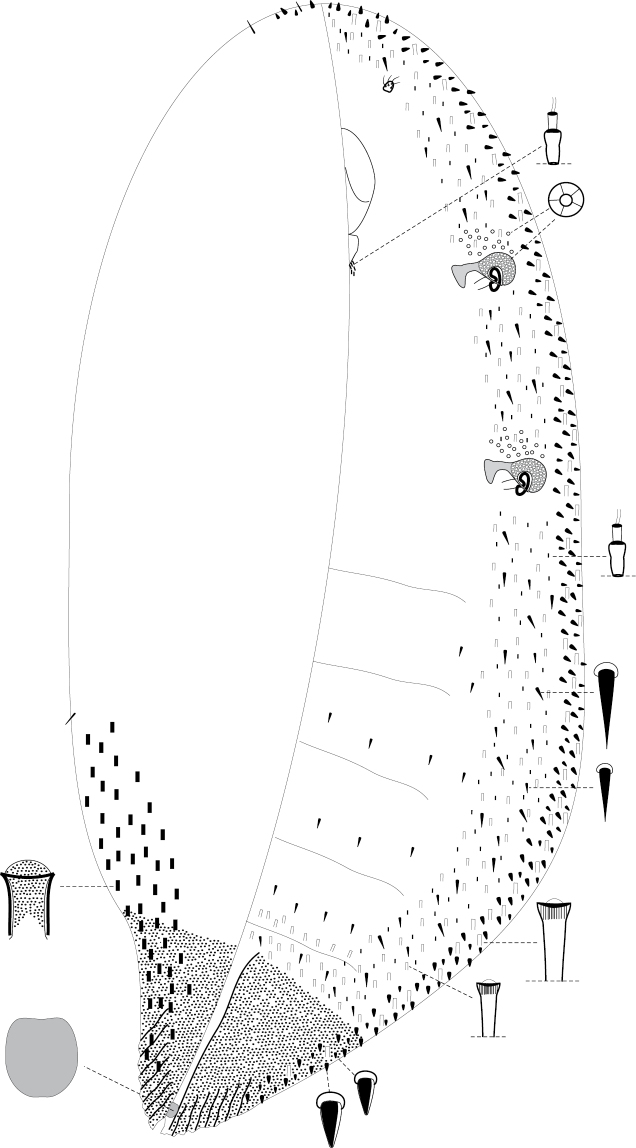
*Aclerda pseudozoysiae* sp. n., holotype.

The scale insect family Aclerdidae currently includes 5 genera with 58 species (ScaleNet <http://www.sel.barc.usda.gov/scalenet/scalenet.htm> – Ben-Dov et al. 2012) distributed mainly in hot and dry, often semi-desert regions of the world. Most of the species are connected with grasses (Poaceae), inhabiting leaf sheathes, they demonstrate very specialized morphological characters such as the absence of legs and strong reduction of antennae, unique anal apparatus, unique invaginated setae, and others (see Fig. 1).

The species of the family have never been specially studied. However, Moharana (1990) noted 2n=18 for undetermined species of *Aclerda* Signoret, 1874 from India without photos or comments on karyotype and genetic system of this species. During an expedition to Indonesia in October – November 2011 I was able to collect two species of *Aclerda*. One of those species is suggested to be new for science and is described below; the second species, *Aclerda takahashii* Kuwana, 1932, is known to be widely distributed in tropical zones of the world. The study of the collected material has provided a possibility to present here some information on cytogenetics and reproductive biology of both discussed species.

## Material and methods

*Aclerda pseudozoysiae* sp. n. K 884, Indonesia, New Guinea (Irian Jaya), vicinity of Jayapura city, slopes of Cyclop mountains above Entrop, dry primary forest interrupted by agricultural crops and sandy burrows, under the leaf sheaths of undetermined grass (Poaceae), 1.XI. 2011, Ilya Gavrilov-Zimin.

*Aclerda takahashii*. K 933, Indonesia, South-Eastern Sulawesi, vicinity of Kendari city near Haluoleo airport, chaotic agricultural plantations after recent deforestation, under the leaf sheaths of *Saccharum* sp., 12.XI.2011, Ilya Gavrilov-Zimin.

All material, including the types of the new species, is preserved in the Zoological Institute, Russian Academy of Sciences, St. Petersburg.

The chromosomal plates were prepared using a squash method in a drop of lactoacetorcein as previously described (Gavrilov and Trapeznikova 2007, 2008).

### Aclerda pseudozoysiae sp. n.

urn:lsid:zoobank.org:act:00A57BC1-DD95-4220-A601-C1554AFEBB87

http://species-id.net/wiki/Aclerda_pseudozoysiae

Fig. 1

#### Adult female.

Body elongate oval, up to 7 mm long, slightly curved. Antennae small, 1-segmented, with several setae. Eyes and legs absent. Spiracles in two pairs; each with large and nearly circular and heavy sclerotized peritrema, covered by numerous quinquelocular pores. Posterior end of body heavily sclerotized on both surfaces even in very young females, abruptly narrowed and acutely pointed, ridged. Anal cleft short, about the same length as anal plate. Form of anal plate shown on the enlargement of Fig. 1. In general, the structure of anal complex is poorly visible because of heavy sclerotization of anal region of body, but it looks like anal complex in other species of the genus. Tubular ducts of 3 sizes: large tubular ducts about 18 µm long; medium-sized ducts about 10 µm long; and microtubular ducts about 7–8 µm long. All 3 types of ducts form ventral submarginal band as shown in Fig. 1. Microtubular ducts form also a group near labium. Quinquelocular pores form small groups near spiracles (with about 10–20 pores in each group). Dorsal invaginated setae (about 12–15 µm long) arranged along submarginal area of abdomen.

#### Taxonomic notes.

The large and widely distributed genus *Aclerda* was comprehensively revised by McConnell (1953). After this review no new *Aclerda* species have been described from Australasian or Indomalasian regions and in view of this I consider the McConnell’ s identification key as correct until now. Based on McConnell’skey, figures and descriptions, *Aclerda pseudozoysiae* sp. n. is similar to *Aclerda zoysiae* McConnell, 1953 which was described from the Philippine Islands, but differs in the presence of 3 types of tubular ducts which are all located on the ventrum only in contrast to *Aclerda zoysiae* having two types of ducts only (microtubular and macrotubular) distributed on both surfaces of the body.

#### Material.

**Holotype:** female, K 884, vicinity of Jayapura, under the leaf sheath of undetermined grass (Poaceae), 1.XI. 2011, specimen in a black circle. Paratypes: 1 female on the same slide; 3 females on other slides and series of unmounted females and larvae in acet-ethanol; all with the same collecting data as holotype.

#### Etymology.

The species name “*pseudozoysiae*”is composed of *pseudo* (false) and “*zoysiae*”, and is intended to show its similarity to the related species, *Aclerda zoysiae*.

##### Cytogenetics and reproductive biology

Both species are ovoviviparous; all stages of embryonic development occur inside the mother’s body. In view of the absence of any notes on ovisacs in other species of *Aclerda* in the coccidological literature, I suppose that the genus as a whole is ovoviviparous.

**Figure 2.**
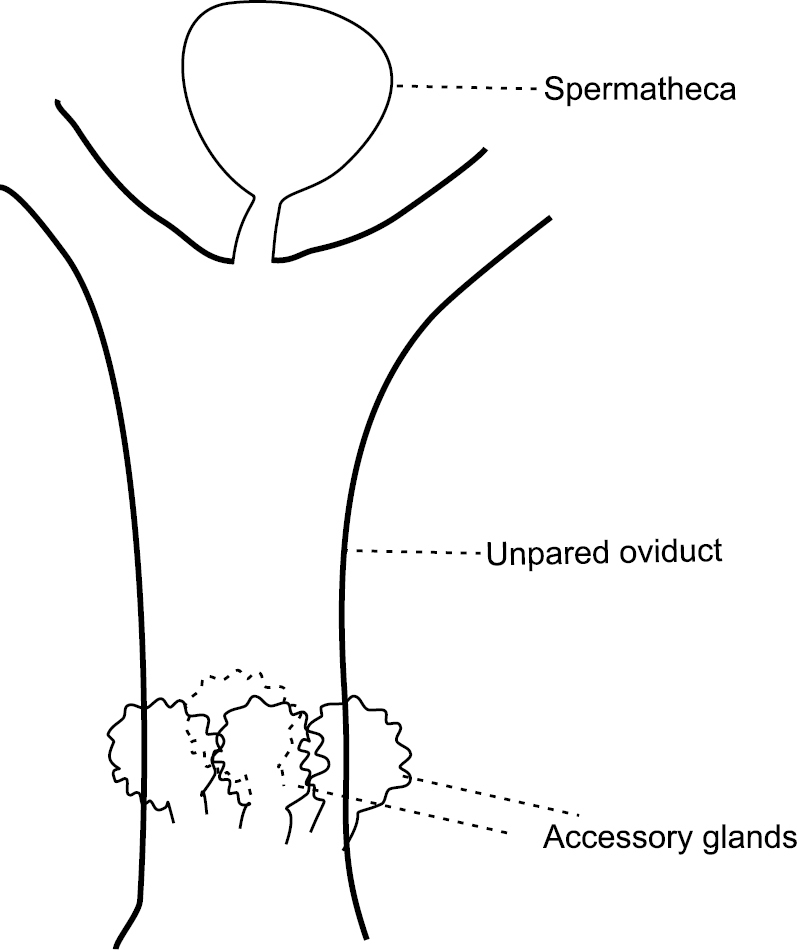
Schematic drawing of oviducts and spermatheca of studied *Aclerda* spp.

Both species have a spermatheca, attached medially between two lateral oviducts (Fig. 2).

Unexpectedly, the mode of reproduction is found to be absolutely different in these two species.

**Figures 3–8.**
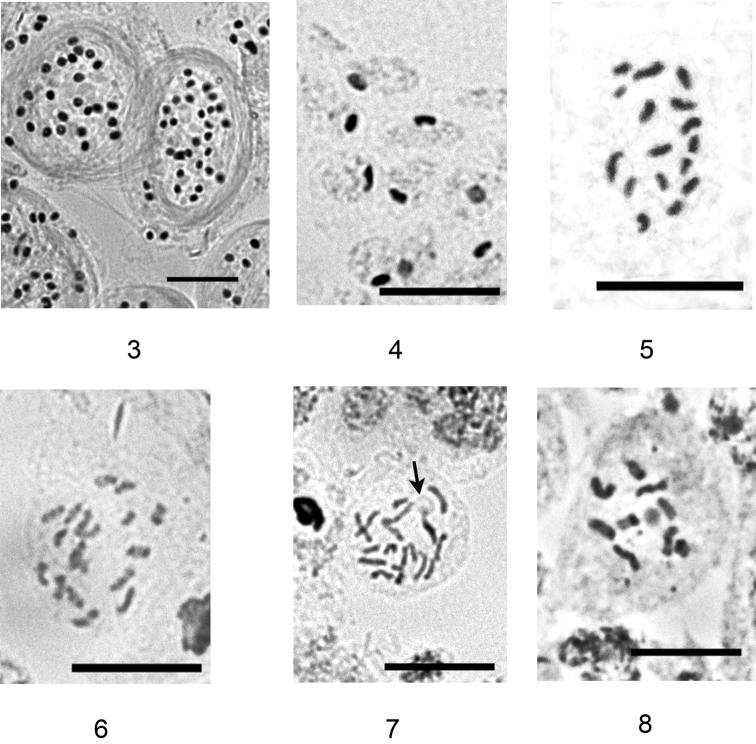
**3–5** *Aclerda pseudozoysiae* sp. n.: **3** bundles of sperms, **4** heterochromatinization of one haploid set of chromosomes (black bodies inside the cells), **5** karyotype **6–8** *Aclerda takahashii*: **6** diploid karyotype, **7** diploid karyotype with nucleolus (arrowed) **8** haploid karyotype. Bar = 10 µm.

*Aclerda pseudozoysiae* has bisexual reproduction, with the presence of male stages of the life cycle in the analyzed population. The studied male ultimonymphs contained bundles of sperms in their testicles (Fig. 3). Specimens with meiotic divisions were not collected. Male larvae and nymphs, and about 50% of the embryos inside each of the four dissected adult females demonstrated a heterochromatinization of one haploid set of chromosomes (Fig. 4), that is common for the majority of cytogenetically studied groups of the superfamily Coccoidea (see, for example, the review of Gavrilov 2007). According to the special experimental studies, elaborated on different genera of Coccoidea (see Brown and Nelson-Rees 1961) the presence of heterochromatinized haploid set characterizes male developmental stages only and moreover, the heterochromatinized set is usually of the paternal origin. Based on this heterochromatinization, *Aclerda pseudozoysiae* is suggested to have either a Lecanoid or a Comstockioid genetic system, these systems being difficult to distinguish without special analysis of male meiosis (see, for example, Nur 1980). The diploid karyotype of *Aclerda pseudozoysiae* includes 16 chromosomes forming gradual size series (Fig. 5).

On the contrary, in the studied population of *Aclerda takahashii*, no male stages of the life cycle were found and adult females did not have sperms and their spermathecae and oviducts. So, the species demonstrates a parthenogenetic form of reproduction. The diploid chromosomal number of *Aclerda takahashii* was found to be 18 (Fig. 6, 7) with chromosomes forming more or less gradual size series. Some of the cells showed a nucleolus located at the end of one of the longer chromosomes (Fig. 7) (the localization of NORs in scale insects was discussed earlier by Gavrilov and Trapeznikova 2007). The heterochromatinization of one haploid set of chromosomes was not found in any of about 150 studied embryos from 4 females and, so, theoretically, all these embryos must be female embryos. However, only about 50 % of the embryos inside each studied female were diploid, and the others demonstrated haploid number (n=9) of chromosomes in each of the cells (Fig. 8). This sudden form of parthenogenesis seems to be unknown in scale insects. Usual haplo-diploidy is inherent in different species of Iceryini scale insects (superfamily Orthezioidea), but diploid progeny are characteristically produced by fertilized Iceryini females only (Hughes-Schrader 1948). In the superfamily Coccoidea, parthenogenesis with different ways of diploidy restoration is known in different families, but in all studied cases of deuterotoky and arrhenotoky, haploid embryos are not produced, diploidy is restored in all embryos and the heterochromatinization of one haploid set of chromosomes marks male embryos (Nur 1971, 1980). Probably, in *Aclerda takahashii* the parthenogenetic diploidy restoration takes place in a part of embryos only. The question whether haploid embryos are able to produce viable males/ females can not be answered without additional observations in the field and laboratory experiments.

## Supplementary Material

XML Treatment for Aclerda pseudozoysiae
